# Catalytically Impaired *TYK2* Variants are Protective Against Childhood- and Adult-Onset Systemic Lupus Erythematosus in Mexicans

**DOI:** 10.1038/s41598-019-48451-3

**Published:** 2019-08-21

**Authors:** Cecilia Contreras-Cubas, Humberto García-Ortiz, Rafael Velázquez-Cruz, Francisco Barajas-Olmos, Paulina Baca, Angélica Martínez-Hernández, Rosa Elda Barbosa-Cobos, Julian Ramírez-Bello, Maria A. López-Hernández, Yevgeniya Svyryd, Osvaldo M. Mutchinick, Vicente Baca, Lorena Orozco

**Affiliations:** 1 Immunogenomics and Metabolic Diseases Laboratory, National Institute of Genomic Medicine, SS Mexico City, Mexico; 2 Servicio de Reumatología del Hospital Juárez de México, Mexico City, Mexico; 3 Unidad de Investigación en Enfermedades Metabólicas y Endócrinas del Hospital Juárez de México, Mexico City, Mexico; 4 Department of Genetics, Instituto Nacional de Ciencias Médicas y Nutrición Salvador Zubirán, Mexico City, Mexico; 5 Department of Rheumatology, Pediatric Hospital Medical Center SXXI, IMSS, Mexico City, Mexico

**Keywords:** Genetics research, Systemic lupus erythematosus

## Abstract

Type I interferon (IFN-I) pathway plays a central role in the systemic lupus erythematosus (SLE) pathogenesis. Recent data suggest that SLE is associated with variants in IFN-I genes, such as tyrosine kinase 2 (*TYK2*), which is crucial in anti-viral immunity. Here, five *TYK2* single nucleotide polymorphisms (SNPs) were genotyped in 368 childhood-onset SLE Mexican patients and 516 sex-matched healthy controls. Allele frequencies were also estimated in four indigenous groups. SLE protection was associated with *TYK2* risk infection variants affecting residually its catalytic domain, rs12720356 (OR = 0.308; p = 0.041) and rs34536443 (OR = 0.370; p = 0.034), but not with rs2304256, rs12720270, and rs280500. This association was replicated in a 506 adult-onset SLE patients sample (OR = 0.250; p = 0.005, and OR = 0.277; p = 0.008, respectively). The minor alleles of both associated SNPs had a lower frequency in Mestizos than in Spaniards and were absent or rare in indigenous, suggesting that the presence of these alleles in the Mexican Mestizo population was derived from the Spaniards. For the first time, we report genetic variants with a protective effect in childhood- and adult-onset SLE Mexican population. Our results suggest that the frequency of *IFN-I* alleles associated with SLE, may have been shaped in populations exposed to infectious diseases for long periods, and this could be an explanation why Native American ancestry is associated with a higher SLE prevalence and an earlier onset.

## Introduction

Systemic lupus erythematosus (SLE) (OMIM # 152700) is an autoimmune disease characterized by a broad spectrum of clinical manifestations and the production of autoantibodies against several nuclear antigens. Over the last decade, genome-wide association studies (GWAS) have identified up to 90 risk loci for SLE in various ethnic populations^1,2^. SLE susceptibility is greater in some ethnic groups, including Africans and Hispanics with an Amerindian origin, which exhibit a higher occurrence of lupus nephritis compared to Caucasian individuals^3,4^. Previous studies also demonstrate that Hispanics have an earlier age of SLE onset and are more likely to develop severe clinical manifestations^5–7^.

A haplotype located on IFN-regulatory factor 5 (*IRF5*) shows one of the strongest associations with SLE (OR = 10.46), and was identified in the Mexican Mestizo population. This haplotype occurs at the highest frequency in Mestizos worldwide, and it was still higher in a Mexican indigenous group^8^. Several studies demonstrate that the type I interferon (IFN-I) pathway plays a central role in SLE pathogenesis, in agreement with the concurrence of elevated IFN-α serum levels in several SLE patients^9,10^. Supporting this relationship, association studies in different populations indicate that IFN-I signaling genes contribute to SLE. Recent data also suggest that SLE is associated with variants in the tyrosine kinase 2 gene (*TYK2*), which is essential for IFN-I signaling and plays an important role in anti-viral immunity. However, association studies show inconsistent effect directions among populations. Sigurdsson *et al*. identified two *TYK2* single-nucleotide polymorphisms (SNPs)—rs2304256: Val362Phe and rs12720356: Ile684Ser—that are associated with decreased SLE susceptibility in patients of Nordic ancestry^11^. Graham *et al*. reported that SLE susceptibility is significantly associated with the rs12720270 *TYK2* SNP in UK families. In the Han Chinese population, a gene–gene interaction between *TYK2* and *IRF5* is associated with SLE susceptibility, suggesting that the combined effect of variants located in IFN genes may also play a crucial role in SLE pathogenesis^12^. These varied observations may reflect differences that rely among populations^13^.

Considering the strong association of IFN-pathway genes with SLE in Mexican patients, here we investigated the contribution of *TYK2* variants to the development of childhood- and adult-onset SLE in the Mexican population. We also determined whether the gene–gene interaction of *TYK2* with the risk haplotype of *IRF5* was associated with this disease.

## Results

### Comparison of the allele and genotype frequencies of the *TYK2* variants among populations

The allele and genotype frequencies of all five analyzed SNPs were in Hardy–Weinberg equilibrium. We compared these allele frequencies with those reported in the 1000 Genomes Project Database, and with those determined in the Mexican indigenous population in this study. The allele frequencies of the genotyped SNPs in the healthy control group were similar to those previously reported among individuals of Mexican ancestry from Los Angeles, CA, USA (MXL). Only polymorphisms rs12720356 and rs280500 significantly differed in frequency between the healthy control group and the Spanish population from the 1000 Genomes Project Database (Iberians, IBS) (Table 1).

**Table 1:** Comparison of *TYK2* minor allele frequencies (MAF) of the Mestizo healthy controls and those of Amerindians of this study, and other populations from the 1000 Genomes Project Database.

SNP	Minor Allele	Mestizo (n = 516)	Amerindians (n = 300)	MXL (n = 124)	IBS (n = 214)	EUR (n = 1006)	EAS (n = 1008)	SAS (n = 978)	AFR (n = 1322)
rs12720356	C	0.02	0.003*	0.02	0.07*	0.09*	0*	0.01	0*
rs2304256	A	0.19	0.14	0.16	0.21	0.26*	0.52*	0.29*	0.09*
rs12720270	A	0.17	0.13	0.14	0.14	0.17	0.5*	0.23*	0.03*
rs280500	G	0.05	0.02	0.08	0.2*	0.17*	0.04	0.2*	0.31*
rs34536443	C	0.021	0.001*	0.02	0.03	0.03	0*	0.01*	0*

Mestizo: Healthy donors from this study. Amerindians: Indigenous from the Nahuatl, Maya, Tarahumara and Zapoteco ethnic groups. MXL: Mexican Ancestry from Los Angeles USA. IBS: Iberian Population in Spain. EUR: European. EAS: East Asian. SAS: South Asian. AFR: African. *p < 0.001 indicates statistical significance.

The variants rs12720356 and rs34536443 were nearly absent in the indigenous population, with frequencies of 0.003 and 0.001, respectively. These frequencies were significantly lower than those observed among the Mexican Mestizo healthy controls (Table 1, and Supplemental Table 1), suggesting that the presence of these alleles in the Mexican Mestizo population was derived from the Spaniards.

### Association of *TYK2* SNPs with childhood- and adult-onset SLE in the Mexican population

Association analysis revealed a protective OR for the C allele of rs12720356 (OR = 0.308; p = 0.041) and for the C allele of rs34536443 (OR = 0.370; p = 0.034), which remained significant after correction for gender and ancestry. The polymorphisms rs2304256, rs12720270, and rs280500 were not associated with childhood-onset SLE (Table 2). The protective association of both rs12720356 and rs34536443 variants was replicated in an adult-onset SLE Mexican patients sample (OR = 0.250; p = 0.005, and OR = 0.277; p = 0.008, respectively) (Supplementary Table 2).

**Table 2:** Frequencies of *TYK2* alleles in childhood-onset SLE patients and healthy controls. *Corrected *p* value.

SNP	Position	Alleles	Minor Allele	Frequency Cases (n = 368)	Frequency Controls (n = 516)	*p* value*	OR 95% CI
rs12720356	10330975	A/C	C	0.007	0.023	0.041	0.308 [0.099–0.95]
rs2304256	10336652	C/A	A	0.169	0.203	0.153	0.8045 [0.596–1.085]
rs12720270	10336760	G/A	A	0.156	0.176	0.382	0.8641 [0.622–1.199]
rs280500	10351402	A/G	G	0.061	0.052	0.476	1.206 [0.719–2.022]
rs34536443	10463118	G/C	C	0.012	0.021	0.034	0.3702 [0.147–0.928]

OR values are corrected by gender and ancestry. OR = odds ratio; 95% CI = confidence interval. *p* < 0.05 indicates statistical significance.

### LD structure of the *TYK2* SNPs in the Mexican population

**Figure 1:**
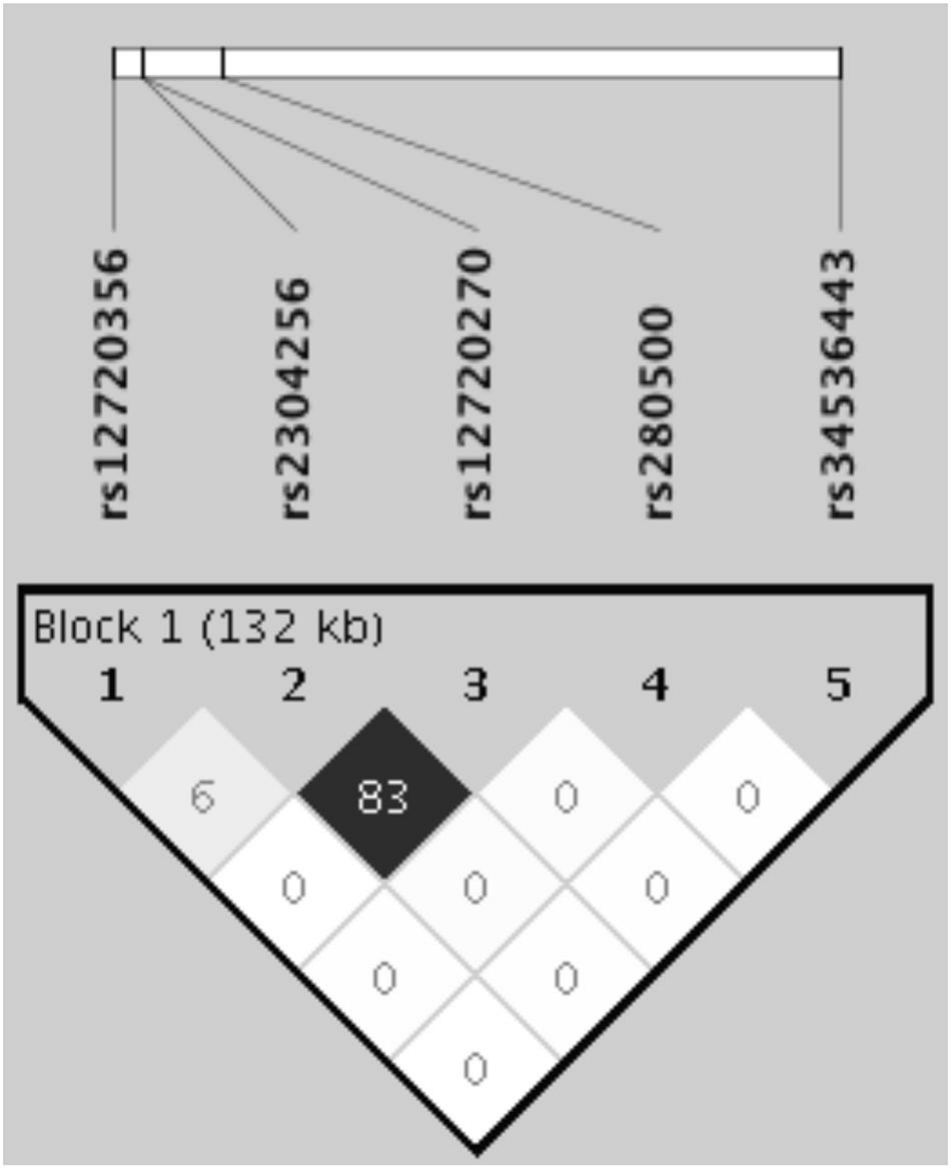
Linkage disequilibrium (LD) plot for the five analyzed *TYK2* polymorphisms. A haplotype block was found for rs2304256 and rs12720270. Black diamond represents the LD, denoted as the r^2^ value.

Haplotype construction revealed five different allele combinations with a frequency of >1% in the Mexican population, two of which were associated with childhood-onset SLE. The CAGAG haplotype, contained the derived alleles rs12720356C and rs2304256A and the three wildtype alleles rs12720270G, rs280500A, and rs34536443G, was associated with protection against childhood-onset SLE (OR = 0.359, p = 0.048). On the other hand, the ACGGG haplotype included only the risk allele of rs280500, and was associated with SLE risk (OR = 1.63, p = 0.034). No other haplotype showed a significant association with SLE (Table 3). LD patterns revealed that only *TYK2* rs2304256 and rs12720270 variants were in LD (r^2^ = 0.83) (Fig. 1).

**Table 3:** *TYK2* haplotypes in Mexican patients with childhood-onset SLE and healthy controls.

Haplotype	Frequency		OR (95% CI)	*p*
	Cases (n = 368)	Controls (n = 516)		
ACGAC	0.013	0.020	0.571 (0.216–1.507)	0.257
ACGGG	0.075	0.045	1.63 (1.036–2.565)	0.034*
AAAAG	0.155	0.158	0.976 (0.729–1.307)	0.87
CAGAG	0.006	0.019	0.359 (0.130–0.991)	0.048*
ACGAG	0.753	0.756	0.996 (0.775–1.281)	0.975

*p < 0.05 indicates statistical significance.

### Gene-gene interactions between *TYK2* and *IRF5*

**Figure 2:**
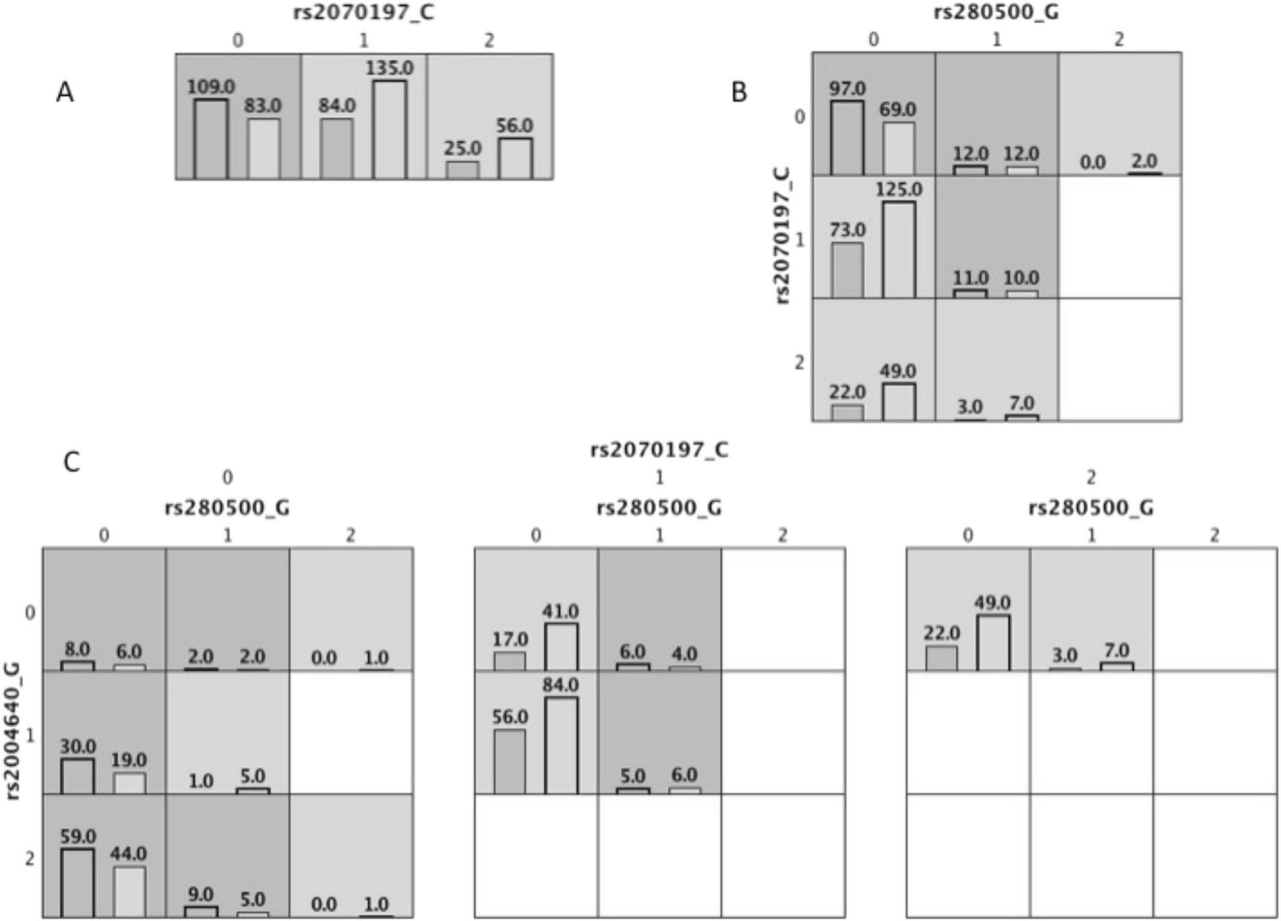
Multifactor dimensionality reduction models of the *TYK2* and *IRF5* interaction. Dark gray columns indicate controls, and light gray columns indicate cases, with the numbers of controls and cases noted at the bottom of each column. (**A**) The best single-locus model for *IRF5* rs2070197/C. (**B**) The best two-locus model, including the *TYK2* variant rs280500/G and *IRF5* variant rs2070197/C. (**C**) The three-locus model, showing interaction between the *TYK2* variant rs280500/G and the *IRF5* variants rs2004640/G and rs2070197/C.

The genotypes of three *IRF5* SNPs, that form the previously identified SLE risk haplotype in the Mexican population, were used for gene–gene interaction analysis between *IRF5* and *TYK2*. Epistasis analysis generated a three-way epistatic model between the *TYK2* rs280500 polymorphism and the two *IRF5* rs2004640 and rs2070197 polymorphisms (Table 4). However, this interaction did not reach statistical significance (TA = 0.617, CV = 8/10, p = 0.184; Fig. 2 and Table 4).

**Table 4:** Results from the best multifactor dimensionality reduction analysis.

Model	SNPs	Genes	TA	CVC	*p*
Single locus	rs2070197/C	*IRF5*	0.598	10/10	0.159
Two-locus	rs280500/G; rs20070197/C	*TYK2*	0.609	9/10	0.149
Three-locus	rs280500/G; rs2004640/G; rs2070197/C	*TYK2* *IRF5*	0.617	8/10	0.184

TA = Testing Accuracy; CVC = Cross-validation Consistency. *p* < 0.05 indicates statistical significance.

## Discussion

The Mexican Mestizo population has a highly heterogeneous genetic structure that mainly comprises Amerindian (56%), European (41%), and a small proportion of African (3%) ancestries^7^. We previously reported a substantial Amerindian contribution to the higher frequency of some SLE risk variants in the Mestizo population—particularly variants in innate immune response genes, such as *IRF5*, which is part of the IFN signaling pathway, and exhibits altered expression in SLE patients^14^. In the present study, we evaluated five variants—rs280500, rs12720270, rs2304256, rs12720356, and the rare functional variant rs34536443—located in *TYK2*, which is also involved in IFN-I and III signaling^15^. Comparing the frequencies of these *TYK2* variants between Mexican Mestizos and IBS revealed significant differences only in rs12720356 and rs280500, which both showed a higher frequency in IBS (0.02 vs. 0.07 and 0.05 vs. 0.2, respectively) (Table 1). To advance our understanding, we further determined the allele frequencies of the *TYK2* variants in four representative Mexican Amerindian groups: Nahuatl, Maya, Tarahumara, and Zapoteco. The minor allele frequencies (MAFs) of these five SNPs were lower in Amerindians than in the Mestizo and IBS populations. Furthermore, the rs12720356 and rs34536443 variants were nearly absent in the indigenous population, with frequencies of 0.003 and 0.001, respectively, and notably both were monomorphic in the Tarahumaras and Zapotecos (Supplemental Table 1). These observations suggest that these alleles in the Mestizo population were derived from IBS.

We also analyzed the contributions of the five *TYK2* SNPs with regard to susceptibility to childhood-onset SLE. In contrast to the observations in Caucasian and Asian populations, the rs2304256, rs280500, and rs12720270 variants were not associated with childhood-onset SLE in the Mexican population (Table 2)^12,13,16^. These discrepancies may be explained by genetic differences in allele frequencies, LD patterns, or gene–gene interactions among populations. In fact, the LD pattern observed for rs2304256 and rs12720270 SNPs among Asian populations and in our present Mexican population (China r^2^ = 0.95, Japan r^2^ = 0.85, and Mexico r^2^ = 0.83) substantially differs from LD patterns reported in Caucasian populations (Sweden r^2^ = 0.40, Finland r^2^ = 0.49, and UK r^2^ = 0.20)^17^. However, we did not identify the gene–gene interactions between *TYK2* and *IRF5* (Table 4 and Fig. 2) that were previously reported in a Chinese population^12^.

Our analysis revealed that the rs12720356 and rs34536443 variants conferred protection against both childhood- (OR = 0.308, p = 0.041; OR = 0.370, p = 0.034, respectively) and adult-onset SLE (OR = 0.250; p = 0.005, and OR = 0.277; p = 0.008, respectively) in Mexican population. These findings are in agreement with previous reports that the rs12720356 polymorphism is significantly associated with protection against SLE in individuals of European ancestry^11,16^. The rs34536443 variant has also been described as protective against other autoimmune diseases, including type 1 diabetes, rheumatoid arthritis, and multiple sclerosis, in other Caucasian populations^11,18^.

Interestingly, rs12720356 and rs34536443 are missense variants. The rs12720356 variant causes an Ile → Ser amino acid change at position 684, which is a pseudo-kinase region JH2 of TYK2. The rs34536443 variant causes a Pro → Ala amino acid change at position 1104, which is located in the C-terminal JH1 catalytic domain of TYK2. Previous reports state that both non-synonymous variants are catalytically impaired but exhibit residual functionality in response to IFN-α/β and other pro-inflammatory cytokines^19^. In contrast to *in vitro* experiments, *in vivo* studies in *Tyk2* kinase-inactive (Tyk2^K923E^) mice suggest that the impaired kinase activity impacts IFN-I signaling through activation of STAT1-4 factors, and alters the viral defense^20^. Thus, these non-synonymous variants might imbalance TYK2 function in the IFN-I pathway, decreasing STAT1-4 phosphorylation, and thus decreasing IFN-I signaling, ultimately counteracting the autoimmune response.

Overall, the presently available data suggest that although most immune genes have been under positive selection in the indigenous population, due to several epidemic episodes after the Spanish conquest, variants within *TYK2*, failing to respond to infections, may have been under negative selection. This may be partly explained by the restricted function of TYK2 in IFN-α signaling. Even though Tyk2-deficient mice respond normally to IL-6 and IL-10 against infections, they require higher IFN-α concentrations to respond in the absence of Tyk2^21^. Nevertheless, they cannot induce IL-12 signaling through STAT4 phosphorylation. This is an effect similar to that found in the B10.Q/J mouse strain, which is characterized by a single missense *TYK2* mutation (E775K) and is strongly sensitive to *Toxoplasma gondii* infection but resistant to experimental collagen-induced arthritis^22^.

Thus, we hypothesize that the scarcity or absence of these SLE-protective alleles in the present-day indigenous populations, may also indicate that these alleles, failing to respond to infections, might had been purged from populations like Native Americans, exposed to endemic infectious diseases for long periods, or negatively selected during the severe epidemics brought by Spaniards during America’s colonization, leading to selection for individuals who could contend against infections. This hypothesis is in line with recent findings, where homozygosity for *TYK2* P1104A confers a predisposition for tuberculosis by a preferential impairment of IL-23-dependent IFN-γ immunity, which could explain the protective effect to SLE, observed in this study. In addition, this observation could be also supported by the fact that the 1104A allele has decreased from 9% to 4.2% over the past 4000 years in Europeans, suggesting that it has been negative selected^23^. Altogether, is in agreement with several studies suggesting that genes involved in the immune response are targets of natural selection in humans, and that certain autoimmune diseases seem to be mediated by these alleles^24,25^.

In conclusion, for the first time, we report two *TYK2* genetic variants with a protective effect in the Mexican childhood- and adult-onset SLE population. These rare variants, rs12720356 and rs34536443, have a lower frequency in Mestizos than in Spaniards and were absent or rare in indigenous, suggesting that the presence of these alleles in the Mexican population was derived from the Spaniards. Thus, the Mexican Mestizos may have inherited higher frequencies of SLE risk alleles from the indigenous population, while protective variants may have been subject to negative selection.

Our results suggest that frequency of *IFN-I* alleles associated with SLE, may have been shaped in populations exposed to infectious diseases for long periods, and this could be an explanation why Native American ancestry is associated with a higher SLE prevalence and an earlier onset.

Several *TYK2* polymorphisms are associated with autoimmune and inflammatory diseases, but studies in different populations show inconsistent results regarding the direction of the effect on SLE susceptibility. This is likely due to the different genetic backgrounds of the studied populations. Studies of variants in genes related to IFN-I signaling in autoimmune diseases are relevant, since such investigations may elucidate key factors in the development of such diseases in certain groups, including the Mexican population. This study brings insights in the genetic complexity of admixed populations such as the Mexican, and highlights the relevance of dissecting variants that may contribute to autoimmune diseases, such as SLE.

## Methods

### Population sample

We performed a case-control association study including 368 patients with childhood-onset SLE and 516 sex-matched healthy blood bank donors, all from Mexico City. All patients fulfilled the American College of Rheumatology (ACR) criteria for SLE diagnosis, as previously described^26,27^. As a replication of associated SNPs with childhood-onset SLE, an independent cohort of 967 individuals from Mexico City consisting of 506 adult-onset SLE patients fulfilling the ACR criteria for SLE diagnosis, and 461 healthy controls, was included. To evaluate the allele frequencies in Native Americans, we also included 300 indigenous individuals belonging to four of the most representative Mexican ethnic groups (Maya, Nahuatl, Tarahumara, and Zapoteco), all of whom were from the previously described Metabolic Analysis in an Indigenous Sample (MAIS) cohort^28^. This study was conducted in accordance with the Declaration of Helsinki. All participants gave their signed informed consent, and the study was approved by the Instituto Nacional de Medicina Genómica local ethics and research committees. Informed consent from a parent and/or legal guardian was obtained for the children study participation, and all included children assented.

### Genotyping

Genomic DNA was isolated from whole blood samples using a Puregene Blood Core kit (Qiagen, Valencia, California, USA). We genotyped five *TYK2* SNPs—including four previously described as associated with SLE (rs280500, rs12720270, rs2304256, and rs12720356) and the rare functional variant rs34536443—using TaqMan SNP Genotyping Assays (Applied Biosystems, Foster City, CA). The results were analyzed using SDS 2.3 software (Applied Biosystems). All SNPs had a call rate of over 98%. To validate the SNP genotyping accuracy, we directly sequenced samples from a randomly selected subset of 15 patients and 15 controls using an automated ABI PRISM 310 Genetic Analyzer (Applied Biosystems, Life Technologies, CA, USA). All sequences showed 100% reproducibility. Ancestry was determined in the discovery cohort using 96 ancestry informative markers (AIMs) contained in a GoldenGate Genotyping Assay (Illumina, San Diego, CA), as previously described^29^. Allele frequencies from the continental populations were obtained from the 1000 Genomes Project Database^30^.

### Statistical analysis

Data from this case-control association study were analyzed by logistic regression using PLINK (v.1.07)^31^. All associations were evaluated under an additive model with adjustment for gender and ancestry. We determined the allele frequencies for cases and controls, the odds ratio (OR), and the 95% confidence interval (95% CI). A *P* value of <0.05 after Bonferroni multiple testing correction was considered to indicate statistical significance. Haploview (v1.0) was used to calculate linkage disequilibrium (LD) through pairwise r^2^ values, to determine allele and haplotype frequencies, and to construct haplotypes^32^. Gene–gene interactions between *TYK2* and *IRF5* were determined using Multifactor Dimensionality Reduction (MDR) software (v.3.0.2.5)^33^.

## Supplementary information

Supplementary Information

## Data Availability

All data generated or analyzed during this study are included in this manuscript.
