# Supplementary material for: Catalytically Impaired TYK2 Variants are Protective Against Childhood- and Adult-Onset Systemic Lupus Erythematosus in Mexicans
Source: Sci Rep. 2019 Aug 21;9:12165. doi: 10.1038/s41598-019-48451-3 (PMC6704113; doi:10.1038/s41598-019-48451-3)
Supplement: Supplementary file 1 — Supplementary Information [file 41598_2019_48451_MOESM1_ESM.pdf]

Title: Catalytically Impaired *TYK2* Variants are Protective Against Childhood- and Adult-Onset Systemic Lupus Erythematosus in Mexicans

Authors: Cecilia Contreras-Cubas[1†], Humberto García-Ortíz[1†], Rafael Velázquez-Cruz[1], Francisco Barajas-Olmos[1], Paulina Baca[1], Angélica Martínez-Hernández[1], Rosa Elda Barbosa-Cobos[2], Julian Ramírez-Bello[3], Maria A. López-Hernández[4], Yevgeniya Svyryd[4], Osvaldo M. Mutchinick[4], Vicente Baca[5] and Lorena Orozco[1]*.

[1] Immunogenomics and Metabolic Diseases Laboratory, National Institute of Genomic Medicine, SS, Mexico City, Mexico

[2] Servicio de Reumatología del Hospital Juárez de México

[3] Unidad de Investigación en Enfermedades Metabólicas y Endócrinas del Hospital Juárez de México

[4] Department of Genetics, Instituto Nacional de Ciencias Médicas y Nutrición Salvador Zubirán, Mexico City, Mexico

[5] Department of Rheumatology, Pediatric Hospital Medical Center SXXI, IMSS, Mexico City, Mexico

*Correspondence: lorozco@inmegen.gob.mx

[†]Equal Contributors

SNP	Minor Allele	Natives	Nahuatl	Maya	Tarahumara	Zapoteco
rs12720356	C	0.003	0.013	0.014	0.000	0.000
rs2304256	A	0.14	0.134	0.276	0.045	0.1153
rs12720270	A	0.13	0.122	0.281	0.043	0.0957
rs280500	G	0.02	0.044	0.027	0.000	0.000
rs34536443	C	0.001	0.002	0.001	0.000	0.000

Supplementary Table 1. Comparison of the *TYK2* Minor Allele Frequencies in four indigenous groups.

SNP	Position	Alleles	Minor Allele	Frequency Cases (n=506)	Frequency Controls (n=461)	*p* value*	OR 95% CI
rs12720356	10330975	A/C	C	0.005	0.022	0.005	0.250 [0.093-0.670]
rs34536443	10463118	G/C	C	0.006	0.024	0.008	0.277 [0.106-0.720]

Supplementary Table 2. Frequencies of *TYK2* rs12710356 and rs34536443 alleles in adult-onset SLE patients and healthy controls. *Corrected *p* value. OR=odds ratio; 95% CI=confidence interval. $p < 0.05$ indicates statistical significance.
